# Cognitive Speech Therapy Protocol directed to Autistic Spectrum Disorder (PROFOCO-ASD): construction stage

**DOI:** 10.1192/j.eurpsy.2024.1495

**Published:** 2024-08-27

**Authors:** M. V. Stlpen, D. R. M. Avejonas, D. C. Dias

**Affiliations:** ^1^REHABILITATION SCIENCES, UNIVERSIDADE DE SÃO PAULO, SÃO PAULO, Brazil

## Abstract

**Introduction:**

Autism Spectrum Disorder (ASD) is considered a neurodevelopmental disorder characterized by changes in cognitive aspects that influence the process of social communication development in these individuals. The Speech-Language Pathologist is the professional qualified to evaluate and intervene in cases of language impairment, however, there are few accessible cognitive assessment instruments. The Cognitive Speech Therapy Protocol (PROFOCO) is a questionnaire to assess cognitive aspects directed to children with a clinical diagnosis of Autism Spectrum Disorder (ASD).

**Objectives:**

The present study aims to present the construction phases of the Cognitive Speech Therapy Protocol aimed at Autistic Spectrum Disorders (PROFOCO-ASD), with emphasis on the panel of experts.

**Methods:**

The Cognitive Speech Therapy Protocol was prepared as a PhD thesis in the area of Rehabilitation Sciences at the Faculty of Medicine of the University of São Paulo (FMUSP). This is a questionnaire to investigate cognitive aspects aimed at children between 2 and 12 years old, diagnosed with Autism Spectrum Disorder, to be applied by a speech therapist and answered by parents or guardians. The construction of the protocol took place in 4 stages: experience of the authors, review of updated literature, a pre-test applied in person to 10 parents and guardians of children with ASD and the panel of experts where the protocol was analyzed by 3 specialists from area of speech therapy linked to USP AND UNIFESP, with experience in language and in the construction of protocols as criteria for selection, which analyzed the content of the questions, the vocabulary, the structure of the protocol and the answer

**Results:**

The authors’ experience in the construction process made it possible to observe the need to introduce issues involving the adequate state of brain regulation, conditions for reception, analysis and storage of information and conditions for programming, regulation and execution of activities. The updated bibliographic review made it possible to elaborate each question based on scientific evidence. The pre-test made it possible to analyze the understanding of the proposed questions, the vocabulary used and the time required for application. The expert panel provided an analysis of the content and vocabulary, leading to relevant changes in its general context, demonstrating the importance of the expert panel phase in the development of a reference protocol.

**Conclusions:**

The present study demonstrates the importance of the expert panel phase in structuring a screening instrument, since a different perspective from people involved in the language area can give more clarity to the questions, as well as the vocabulary used, ease of A protocol capable of being understood by a population in different contexts, its vision also evolves, in different regions.

**Disclosure of Interest:**

None Declared

